# Training New Doctors in Mozambique. A Sustainable International Aid Health Program

**DOI:** 10.3390/ijerph18031355

**Published:** 2021-02-02

**Authors:** Manuel Romero-Hernández, Patricia Barber, Coraima Clavijo-Sánchez, Luis López-Rivero

**Affiliations:** 1 Department of Business History and Social Sciences, University of South-Eastern Norway, 3199 Borre, Norway; 2 Department of Applied Economic Analysis, University of Las Palmas de Gran Canaria, 35017 Las Palmas de Gran Canaria, Spain; coraima.clavijo101@alu.ulpgc.es; 3 Department of Quantitative Methods in Economy and Management, University of Las Palmas de Gran Canaria, 35017 Las Palmas de Gran Canaria, Spain; patricia.barber@ulpgc.es; 4 Department of Medical and Surgical Sciences, University of Las Palmas de Gran Canaria, 35016 Las Palmas de Gran Canaria, Spain; luismateo.lopez@ulpgc.es

**Keywords:** economic evaluation, human capital, cost-effectiveness, healthcare, higher education, JEL codes D61, I15, I230, I250, and O12

## Abstract

The collaborative project between the University of Las Palmas de Gran Canaria and the public University of Mozambique, UniZambeze, aims to strengthen the institutional and training capacities of its Faculty of Medicine located in Tete to provide new medical graduates. The essence of the program, training doctors, has the objective of improving the healthcare system and making it sustainable for the integration of new graduates into the staff of the faculty. In this work, we determine the cost of education for a new doctor and we evaluate the social benefit of the program in terms of the human capital. The program has led to the training of 199 new doctors in the 11 years of operation and is leading the way for 100 more in the next four years. The incorporation of some of them into the faculty’s staff will generate new doctor graduates in the near future with a cost below 6000 EUR each in normal circumstances. These results can help to determine how much traditional international aid healthcare programs can save when investing in the education of new doctors. This program is an alternative policy for the international aid financing budgets of donor countries. Supporting teachers and native doctors in the future with private and public patronage programs can raise the efficiency per EUR spent.

## 1. Introduction

The essence of the program between the University of Las Palmas de Gran Canaria (ULPGC) and the UniZambeze, training doctors, has the objective of improving the healthcare system in Mozambique and making it sustainable for the integration of new graduates into the staff of the Faculty of Medicine in Tete who will substitute the presently foreign teachers in the future. The project complies with the two goals proposed by the new millennium declaration of the United Nations to reduce poverty in developing countries through health and education. The program is in line with what the World Health Organization considers a priority for cooperation in Mozambique, strengthening and addressing workforce education [1]. The international experience of healthcare aid programs in sub-Saharan countries involving universities is linked to postgraduate programs such as Primary Health Care and Family Medicine Education [2] or pre-graduate programs [3]. The program described in this work is fully integrated into the official graduate program of the UniZambeze.

Mozambique has a population of 25.8 million people with a life expectancy of 57.7 years old, which is below the average for African countries (average, 59.6). The neonatal mortality rate is 27.79 per 1000 before the 28th day of life (average, 27.16). Chronic malnutrition affects 41% of children under five. Mortality under five is 73.22 (average, 76), with a maternal mortality ratio of 289 per 100,000 live births. Furthermore, 47.15% of the population is under 15 years old, and 70% of the population lives in rural areas. The illiteracy is 60%, and the gross domestic product (GDP) per capita is 387 USD. The health expenditure per capita is 13 USD. Lastly, 54.7% of the population lives under the umbrella of poverty [4].

Whereas Africa has an average of 2–3 health workers per 1000 people [4], Mozambique has 0.03 doctors and 0.21 nurses per 1000 inhabitants, which is considered in a critical zone for health. The malaria incidence is 337.92 per 1000 people at risk. The tuberculosis incidence rate is 551 in comparison to the average of 230.59 in Africa. The human immunodeficiency virus (HIV) incidence is 10.1 per 1000 uninfected people. The main disease prone to epidemics is cholera, whereby acute respiratory tract infections and diarrheal diseases are the common causes of death and illness [4].

Healthcare is provided by the public sector, private companies, nongovernmental organizations, and religious entities. The country has 1277 healthcare centers distributed across four levels with 15,877 beds and 26,000 health workers. Level 1 offers essential services, urban and rural healthcare centers, and essential healthcare spots. This represents 96% of the healthcare centers in the country. Level 2 involves district hospitals that represent the reference for first aid care; they can attend to emergencies, lower and obstetric surgeries, and simple traumas. Levels 3 and 4 constitute eight province hospitals and four central and specialized hospitals, two in Maputo, one in Beira, and one in Nampula [5]. The geographical framework of this work involves the Province of Tete in the central part of Mozambique, where in 2009, there were 100 doctors, one surgeon, one gynecologist, two internists, and one pediatrician for a population of two million.

In this paper, we present an economic evaluation determining, first, the cost performance of the Faculty of Medicine and the cost of education for a new doctor, as well as how both have developed with the implementation of the program. Second, we calculate the social benefit of the program in terms of the human capital created. Determining the cost performance of the faculty has also made it possible to anticipate the cost of training new doctors in the future when the faculty will be able to work with its own staff. After adjusting for salaries, these results could be applied to other countries with social and health conditions similar to Mozambique. This can help international aid agencies to identify new alternatives to carrying out healthcare programs focused on the education of new doctors and the costs required to develop them.

Section 2 of this work describes and explains the methodology applied, the program, and the organization of the Faculty of Medicine under study. Section 3 presents the results obtained in terms of cost per graduate and how this has developed during the project’s execution. Moreover, this section contains an evaluation of social benefits in terms of the human capital. Lastly, discussion and conclusions are offered in Section 4 and Section 5.

## 2. Materials and Methods

At an international conference in Gran Canaria in 2010, the rector of the UniZambeze requested the support of specialists who could teach the second cycle as part of the Faculty of Medicine in Tete. Two years later, the first 13 teachers of the ULPGC moved to teach in the second cycle of the training program. In 2019, 199 new doctors graduated. Since the program was completed in 2020, the Faculty of Medicine has been functioning entirely with its own staff.

The academic year at the Medical School of Tete runs from February to November in two semesters. The training process for new graduates takes seven years of studies, six years in the school and one year of rotation. Rotations involve the practical application of theory by students in hospitals and healthcare centers supervised by a doctor. The training program started in 2009 with 49 students enrolled in the first year. As of November 2019, more than 1000 students were enrolled in the process of training. During this period, the faculty staff was made up of Mozambicans, Cubans, and ULPGC teachers.

Financed by both private and public Spanish institutions, between 2012 and 2019, more than 75 teachers migrated from the ULPGC to Tete. They taught 13,400 h of classes in periods of four and six weeks, depending on the specific teaching plan. During this time, five cohorts of students graduated, and the new doctors integrated into the public and private Mozambican healthcare system. As the Faculty continues working, the number of doctors graduating will continue to rise in the future.

The faculty started with 21 Mozambican teachers staffing the first cycle. In the subsequent years, as the number of students increased, so did the number of instructors, firstly with Mozambicans and later with Cubans. In 2011, the first six Cuban teachers were incorporated to complete the template. In 2013, coinciding with the beginning of the fifth class of graduates, this number rose to 10. This number remained high until 2016, at which point the new graduates were incorporated as teachers into the Faculty, substituting Cubans. The philosophy of the program was to make the Faculty sustainable and be able to train new physicians internally. In 2018, the number of Cuban teachers was reduced to only two.

There is extensive literature discussing how to measure costs of medical education through the use of cost-effectiveness calculations and analyses [6,7,8,9,10,11,12,13,14,15,16]. In 2007, the World Health Organization published [17] the definition of health workers, together with the costs and impact of having them in developing countries.

Until 2019, the second cycle was exclusively covered by Spanish teachers; however, when the aid program finished in 2020, four Cuban teachers taught in the second cycle that year for the first time. In 2020, the second part of the program of the ULPGC started at the University Hospital of Beira, training new specialists. Some of these specialists are expected to substitute the Cuban teachers in the next four years, making the training process completely independent in the future.

In the Faculty template, there are permanent and part-time teachers. Every permanent teacher provides 248 h of lectures per year, whereas part-time teachers provide up to 8 h a week. Cuban teachers are all permanent. For the last 11 years, 43,200 h were taught, with 28,800 h in the first cycle and 14,400 h in the second cycle. In 2020, by the end of the program, Spanish teachers will have taught 14,400 h. While the annual salary of a Mozambican teacher is 6000 EUR, the salary of a Cuban teacher is 28,500 EUR per year (2019 prices), equating to 24.19 and 114.92 EUR per hour, respectively.

The teachers of the ULPGC did not receive any salary for their activity at Tete’s School of Medicine. Their travel expenses and insurance were paid for by the aid program, while the UniZambeze supported the expenses of an assistant and the house where they stayed in Tete. Considering all those expenses, the average cost for each Spanish teacher in Tete was 1698 EUR. Each Spanish teacher visited Tete once a year. Following [18], we added a second scenario to the analysis of the cost performance of the Faculty, for which we assigned a cost of opportunity to the time of the Spanish teachers. We assumed this cost as being equivalent to the salary that they would receive for that period of time working in Spain, i.e., 4500 EUR per month. As of 2015, following the stabilization of the number of the Spanish teachers, the annual cost in the second cycle was fixed at 72,648 EUR annually, with an hourly cost of 37.8 EUR per lecture. Considering the wage of Spanish teachers, the hourly cost was determined to be 112.8 EUR.

The collaboration between the ULPGC and the UniZambeze had effects firstly on the economic performance of the Faculty of Medicine in Tete, making it sustainable, as well as reducing the cost of education for new doctors. Second, there have been effects on the healthcare system. The third benefit is the contribution to the formation of the human capital of the country. In this paper, we only focused on the measurement of the cost performance of the Faculty of Medicine and on the estimation of social benefit derived from the increase in the human capital.

Through an analysis of the cost performance and indicators of cost efficiency of the Faculty in terms of teaching resources, we were able to study how the efficiency has developed in terms of the performance of the Faculty per student enrolled and graduated as well as make an estimation of its expected development in the future, both in the short term, when the Spanish teachers leave the second cycle, and in the long term, when the Mozambicans become able to teach in the second cycle, definitively substituting the Cubans teachers.

We also calculated how the implementation of the program has developed the cost per student, both enrolled and graduated, for every class of graduates. Differentiating between the first and the second cycle, we were able to measure how the incorporation of new graduate students as teachers in the Faculty changed the efficiency and cost per student enrolled and graduated and how this cost has developed with the composition of the staff. The calculation of the incremental cost-effectiveness ratio (ICER) reports how much the cost of teaching per student has increased, how it is expected to develop in the future, and how the incorporation of new Mozambican teachers benefits the cost-effectiveness per student.

The socioeconomic implications of higher education are often assessed using the human capital method when considering the educational institution as a producer of individuals who acquire competences, and this contributes to the economy by increasing the productivity [19,20,21], which will have additional benefits for the society in the long term. The relationship between human capital formation, economic development, social welfare, and democracy is extensively addressed in the literature [22,23,24,25,26,27].

In this paper, we approximate the social benefit generated by the increase in the human capital as the difference, for each student graduating during their working life, between the income expected working as a doctor after completion of their higher education at the Faculty (10,200 EUR, 2019 prices) and what they would receive working in the labor market with a lower qualification, i.e., the average salary [28].

## 3. Results

The highest total annual cost for the first and second cycles was over 494,000 EUR in 2014, coinciding with the maximum number of Cuban teachers recruited: 294,000 EUR for the first cycle and 67,554 EUR for the second cycle. Later, the replacement of Cuban teachers by Mozambicans reduced the cost of teaching in the first cycle. In 2019, the complete first cycle was covered only by Mozambican assistant teachers with a total annual cost for the first cycle of 69,677 EUR, whereas it was 152,407 EUR in the second cycle, including the salaries of Cuban teachers.

### 3.1. Cost Performance

Table 1 shows the total cost per class of graduates considering the situations with and without a salary for the Spanish teachers, along with the cost per student enrolled and graduated. The highest cost corresponded to the fifth class of graduates with a total cost of 359,000 EUR without salaries and 503,825 EUR upon assigning a cost of opportunity for the time of the Spanish teachers. This high cost also coincided with the highest number of hours taught by Cuban teachers in the first cycle.

As a consequence of the substitution of Cuban teachers, the savings in terms of the total cost between classes of graduates eight and seven was around 46%. The overall cost savings in the program budget became positive for classes of graduates six and seven. With only Mozambicans teaching, the cost of the first cycle for class of graduates nine was the lowest ever achieved, 107,177 EUR, which is also the level expected for subsequent classes of graduates. This reflects the success of the program in terms of the total cost of performance of the Faculty. However, there was a cost increase in the second cycle for class of graduates seven as a consequence of the Spanish teachers leaving the program, who were substituted by Cuban teachers. This will also be mitigated in the future, as soon as the new Mozambican specialists are fully trained and can incorporate into the staff of the Faculty.

The minimum total cost per graduate student achieved for one of the five classes of graduates was 5368 EUR, coinciding with a higher number of students graduated and the increasing presence of Mozambican teachers as previously indicated. The cost per student for the fifth class of graduates, 13,839 EUR, presents a large deviation for several reasons. There was a high number of Cuban teachers initially contracted, while the number of students enrolled and graduated fell considerably for that class (26 students). Since class of graduates seven, the number of students has reduced progressively due to a decision by the government arguing that they could not afford new doctors in the public sector due to their budget.

The number of students reached the maximum in class of graduates seven (79 students), while also achieving the maximum level in the Faculty in terms of total students. This will have consequences in later years in relation to the cost per student graduated until the effect of the government’s decision comes into play.

Table 2 shows the cost increase per enrolled and graduated student in 2015 by class of graduates, globally and for the first and second cycles. The ICER was calculated by dividing the cost difference between the outputs. The interpretation of the ICER results, measuring the efficiency per student enrolled and graduated, may be contradictory for this project because they are strongly influenced by the decrease in the number of students and the simultaneous rise in the cost of Cuban teachers. After class of graduates six, once the number of students rose again and the program became effective with the new graduates substituting the Cuban teachers, the ICER figures showed the benefits on the cost performance of the Faculty.

Table 3 shows the benefit of the efficiency as a percentage. A positive value demonstrates that the difference between promotions Pi and P1 was larger than the difference in cost between P2 and P1 per student enrolled. Again, the political decision to reduce the number of students enrolled has seriously affected the efficiency and cost of a new graduate. The ICER per graduate student shows that even if the first class of graduates had the lowest cost, a decreasing tendency in the cost difference per class of graduates is present. We can see that the number of students that graduated decreased in 2019 and the ICER presented a negative value, denoting no reduction in cost for this class of graduates. Positive values indicate cost savings; therefore, the last three classes of graduates achieved important benefits with respect to the first two classes of graduates. However, in the second cycle, the effect of the Spanish teachers leaving the program can be noted.

Class of graduates 11 will be the first to be taught completely by Mozambicans in the first cycle and by Cubans in the second. This is a good representation of future scenarios in the short term at the faculty, at least in the four years after 2020, until new Mozambican specialists are trained and can be part of the staff of the Faculty. In this scenario, Table 4 shows the cost per student enrolled and graduated (assuming that 50% of students who enrolled graduate). In these conditions, the cost per enrolled student varies between 11,613 EUR if 25 students are enrolled and 2993 EUR if 90 students are enrolled. The cost per graduate would vary between 22,332 and 6451 EUR in the same conditions.

The long-term scenario was analyzed by assuming only Mozambicans would be teaching in both cycles, but with 50% extra salaries for teachers in the second cycle. In that case, the cost per enrolled student varies between 5574 EUR if 25 students are enrolled and 1548 EUR if 90 students are enrolled. This cost would be for the whole training program. The cost per graduate would vary between 10,719 and 3096 EUR in the same conditions.

### 3.2. Social Benefit of Human Capital Investment

By assuming that the new doctors do not receive the training program, i.e., they would be receiving the average salary for a professional [21,29], we were able to calculate the gross social benefit in terms of the human capital as the difference between what students would earn from wages working as a doctor after completion of their higher education in the Faculty (10,200 EUR, 2019 prices) and what they would earn if they had stayed in the labor market, i.e., the average salary (676.8 EUR, 2019 prices [29]) in Mozambique. Assuming 40 years of professional dedication and a rate of discount of 3%, the gross social benefit for an individual medical graduate would be around 271,000 EUR for their professional life. By deducing the total cost of teaching, with respect to 2019 prices, the net social benefit of the human capital per class of graduates would range from 7,119,884 EUR for the first class of graduates to 14,737,578 EUR for the fifth class of graduates (we calculated the net social benefit of medical training per class of graduates as a result of the difference between the gross social benefit and the total cost of teaching; students graduate in January) (Table 5). The total net social benefit for the first five classes of graduates would be 47,947,988 EUR. It should be borne in mind that this profitability applies only to the first five classes of graduates and if the Faculty keeps functioning and, thus, new students graduate every year going forward.

Table 6 shows the social benefit for future classes of graduates with the scenarios involving the number of students enrolled and graduated previously analyzed.

## 4. Discussion

The execution of the program has made it possible to train a new graduate in medicine for a little more than 5300 EUR by combining native and foreign resources. A total of 199 new doctors have been trained in the 11 years of intervention. An additional 100 are expected in the next four years. Although this represents a significant financial effort made by the Mozambican government, in the province of Tete, where there have previously been approximately 100 doctors for two million people, this represents an important impact for the healthcare system. The incorporation of some graduates into the Faculty staff will consolidate the sustainability of the system, as well as make it possible to educate new doctors with a cost below 6000 EUR in the future under normal circumstances and a cost of 3000 EUR in the optimistic long-term scenario.

The collaboration program with the UniZambeze ended in 2020; thus, because the training in the second cycle of medical schools in Mozambique must be provided exclusively by specialist doctors, the work of Spanish teachers was substituted by Cubans, thereby increasing the cost of teaching. The new program aimed at training specialist doctors started in 2020, involving the Catholic University of Mozambique and the University Hospital of Beira. This step will be decisive in completely substituting the Cuban teachers, thereby closing the circle and rendering the Faculty completely sustainable and independent.

The results of the program have been affected by political decisions during its execution. Once the program began achieving its goals, the Mozambican government decided that it would not be possible to support so many new doctors in the public sector. They decided to cut the number of students enrolled in the Faculty, thus affecting the cost per new doctor and student enrolled and, as a consequence, the efficiency of the project. This external political decision has had strong effects on the efficiency of the program. As the training of students in the Faculty is an important fixed component, and as the number of teachers required to provide teaching remains the same for each course, the cost per enrolled student has increased. However, the cost of teaching 25, 50, 75, or even 90 students in the same room remains fixed, which exposes the political decision of reducing the number of students enrolled as being unreasonable according to the costs involved. It would be desirable for this decision to change in the future, thereby maintaining the cost of education for a new doctor close to 3000 EUR in the long term.

The social benefit generated in terms of the human capital will also be affected by the number of students. In the long term, the social benefit expected varies between 2.8 million EUR and 11.5 million EUR per class of graduates for the upper and lower ends of the spectrum in terms of the number of students. The rate of return per class of graduates would be over 120% depending on the class graduated, which can be considered very high in comparison to that expected for higher education in low-income countries (around 27%) [29].

This paper has a limitation in that it does not evaluate the social benefit for the Mozambican healthcare system. Furthermore, the benefit calculated in terms of the human capital cannot be used to substitute this parameter. The social benefit for the human capital must be considered as an additional benefit of the project that would have an impact on the economy and the life conditions in Mozambique. Carrying out a cost–benefit analysis of the effects of this project on the healthcare system would be very complex. In addition to the difficulties in gathering information on treatments received by the population, the project has had a huge impact on the number of new physicians in the country who have found many different occupations, e.g., as teachers in the Faculty or as physicians in primary healthcare systems. Some have continued with their education as specialists at the University Hospital of Beira, while some have become part of nongovernmental organizations and some are working in the private sector. 

This aid program can be used as an alternative policy for the international aid financing budgets of donor countries. Supporting teachers and native doctors can raise the efficiency per EUR spent with private and public patronage programs in the future. The MEPI program grant of the United States (US) government represents one such example obtaining positive results [7,19].

## 5. Conclusions

The collaborative program between the ULPGC and the UniZambeze has generated a system that provides university teachers to the Mozambique education system and doctors to the healthcare system. Furthermore, the cost of education has been reduced, thereby promoting the access of new students that will sustainably provide new doctors to the healthcare system.

The economic evaluation presented in this paper allows approximating the efficiency of such programs in training doctors compared with the cost of supporting physicians from a developed country working in a traditional aid program. Governments and nongovernmental organizations can use this as a reference to determine the efficiency of patronage programs for new students, teachers, and doctors, as a complementary alternative in the short and medium term which, in the long term, could be able to substitute traditional healthcare aid programs, i.e., teaching one how to fish instead of giving them a fish.

This type of international aid collaboration generates long-term results; however, it cannot substitute short-term direct aid programs because the African population also currently needs help. This program provides a lower dependency on foreign human resources in the future, thereby extraordinarily improving the availability and regularity of healthcare for the population. The training of new national doctors allows the replacement of foreign doctors, mainly Cubans and Koreans, which also implies a higher cost for the country. The use of local employees for subsequent generations will allow developing the local economy and improving the country’s trade balance.

The program presented in this paper has enabled the generation of new doctors in Mozambique, who will continue training and graduating future students. This type of program not only contributes to the education and healthcare system, but also has an important effect on building social and human capital, particularly in a country with the level of poverty seen in Mozambique. Thus, in the long term, the financing and execution of this type of program in higher education will produce additional benefits for the society in terms of economic and sociopolitical development of the country.

## Figures and Tables

**Table 1:** Cost (EUR, 2019 prices) and number of students per class of graduates (P).

Class of Graduates	P1 2009–2015	P2 2010–2016	P3 2011–2017	P4 2012–2018	P5 2013–2019	P6 2014–2020	P7 2015–2021	P8 2016–2022	P9 2017–2023	P10 2018–2024	P11 2019–2025
Global cost (EUR)											
Without salary	167,615	212,798	305,627	338,174	359,825	307,325	260,585	-	-	-	-
With salary	251,615	310,798	445,627	484,174	503,825	451,325	404,585	-	-	-	-
First cycle cost (EUR)											
Mozambicans	57,677	45,677	25,677	17,677	11,677	25,677	39,677	53,677	59,677	-	-
Cubans	57,000	142,500	209,000	247,000	275,500	209,000	142,500	76,000	47,500	-	-
Total	114,677	188,177	234,677	264,677	287,177	234,677	182,177	129,677	107,177	-	-
Second cycle cost (EUR)											
Without salary	52,938	53,121	70,950	73,497	72,648	72,648	78,408	-	-	-	-
With salary	136,938	151,121	210,950	219,497	216,648	216,648	222,408	-	-	-	-
Number of students enrolled											
First cycle	49	62	64	60	56	71	97	57	38	25	25
Second cycle	49	62	64	30	50	65	76	34	0	0	0
Total	49	62	64	45	53	68	87	46	38	25	25
Graduated	27	31	52	63	26	-	-	-	-	-	-
Cost per student enrolled											
First cycle	2340	3035	3667	4411	5128	3305	1878	2275	2820	-	-
Global without salary	3421	3432	4775	7515	6789	4519	3013				
Global with salary	5135	5013	6963	10,759	9506	6637	4677	-	-	-	-
Cost per student graduated											
Global without salary	6208	6864	5877	5368	13,839	-	-	-	-	-	-
Global with salary	9319	10,026	8570	7685	19,378	-	-	-	-	-	-

**Table 2:** Incremental cost-effectiveness ratio (ICER) per student enrolled in the first and second cycles using class of graduates one as the baseline. ULPGC, University of Las Palmas de Gran Canaria.

	P2–P1	P3–P1	P4–P1	P5–P1	P6–P1	P7–P1	P8–P1	P9–P1
ICER per number of students enrolled								
Global without salary	3476	9201	−8977	48,053	7353	2479		
Global with salary	4553	12,934	−58,140	63,053	10,511	4079		
ICER per student enrolled (first cycle)								
Mozambicans	−923	−2133	−3636	−6571	−1455	−375	−500	−182
Cubans	6577	10,133	17,273	218,500	9500	3167	2375	864
Total	5654	8000	13,636	43,125	6316	1800	1875	682
ICER per student enrolled (second cycle)								
ULPGC without salary	14	1201	−1082	19,710	1232	943		
ULPGC with salary	1091	4934	−4345	79,710	4982	3166		
ICER per student graduated								
Global without salary	1.82	0.64	0.50	−0.11				
Global with salary	1.59	0.64	0.41	−0.07				

**Table 3:** Benefit of the efficiency per student enrolled and graduated (difference between promotions Pi and P1 presented as a percentage).

	(P3–P1) − (P2–P1)	(P4–P1) − (P2–P1)	(P5–P1) − (P2–P1)	(P6–P1) − (P2–P1)	(P7–P1) − (P2–P1)	(P8–P1) − (P2–P1)	(P9–P1) − (P2–P1)
Benefit per student enrolled							
Global without salary	164.7	−358.3	1282.6	111.6	−28.7		
Global with salary	184.1	−1377.1	1285.0	130.9	−10.4		
First cycle							
Mozambicans	131.1	293.9	611.9	57.6	−59.4	−45.8	−80.3
Cubans	54.1	162.6	3222.2	44.4	−51.9	−63.9	−86.9
Total	41.5	141.2	662.8	11.7	−68.2	−66.8	−87.9
Second cycle							
ULPGC without salary	8430.3	−7786.7	13,991.64	8651.0	6601.3		
ULPGC with salary	352.3	−498.3	7206.1	356.6	190.2		
Benefit per student graduated							
Global without salary	−64.6	−72.3	−106.0				
Global with salary	−59.7	−74.2	−104.4				

**Table 4:** Cost (EUR, 2019 prices) and number of students per class of graduates in short- and long-term scenarios.

	Short-Term Scenario				Long-Term Scenario			
	Case 1	Case 2	Case 3	Case 4	Case 1	Case 2	Case 3	Case 4
Global cost (EUR)	290,323	290,323	290,323	290,323	139,355	139,355	139,355	139,355
First cycle cost (EUR)	69,677	69,677	69,677	69,677	69,677	69,677	69,677	69,677
Second cycle cost (EUR)	220,645	220,645	220,645	220,645	69,677	69,677	69,677	69,677
Number of students enrolled	25	50	75	90	25	50	75	90
Number of students graduated	13	25	38	45	13	25	38	45
Cost per student enrolled	11,613	5806	3871	2993	5574	2787	1858	1548
Cost per student graduated	22,332	11,612	7640	6451	10,719	5574	3667	3096

**Table 5:** Social benefit per class of graduates (EUR, 2019 prices).

	Global Cost		Gross Social Benefit	Social Benefit without Salary	Social Benefit with Salary
	Without Salary	With Salary			
P1 2009–2015	188,653	283,195	7,308,537	7,119,884	6,836,689
P2 2010–2016	232,531	339,618	8,059,012	7,826,481	7,486,863
P3 2011–2017	324,240	472,766	12,977,216	12,652,976	12,180,210
P4 2012–2018	348,319	498,700	15,085,898	14,737,578	14,238,878
P5 2013–2019	359,825	503,825	5,970,849	5,611,069	5,107,243
Total	1,453,568	2,098,104	49,401,557	47,947,988	45,849,884

**Table 6:** Social benefit per class of graduates in the future (EUR, 2019 prices).

	Global Cost		Gross Social Benefit	Short-Term Social Benefit	Long-Term Social Benefit
	Short-Term Scenario	Long-Term Scenario			
Case 1	326,761	326,761	3,518,925	3,192,165	2,865,404
Case 2	326,761	326,761	6,767,164	6,440,403	6,113,643
Case 3	326,761	326,761	10,286,089	9,959,329	9,632,568
Case4	326,761	326,761	12,180,895	11,854,135	11,527,374

## Data Availability

The data presented in this study are available on request from the corresponding author.

## References

[1] World Health Organization (2007). WHO Country Cooperation Strategy 2009–2013. Mozambique.

[2] Flinkenflogel M., Essuman A., Chege P., Ayankogbe O., Maesenner J. (2013). Family medicine training in sub-Saharan Africa: South–South cooperation in the Primafamed project as strategy for development. Fam. Pract..

[3] Moodley K., Akintobi T., Fish T., Blumenthal D. (2018). A Pipeline Program to Address the South African Crisis in Human Resources for Health. Ann. Glob. Health.

[4] World Health Organization (2017). Global Observatory Data. https://aho.afro.who.int/data-and-statistics/af.

[5] Ministério de Saúde (MISAU)-Departamento de Planificaçao (2013). Plano Estratégico do Sector da Saúde PESS 2014–2019.

[6] Dauphinee W.D. (2012). Educators must consider patient outcomes when assessing the impact of clinical training. Med. Educ..

[7] Kieran W. (2014). Costs in medical education: How should we report them?. Med. Teach..

[8] Lehmann U., Sanders D. (2007). Community Health Workers: What Do We Know about Them?.

[9] Mccord G.C., Liu A., Singh P. (2013). Deployment of community health workers across rural sub-Saharan Africa: Financial considerations and operational assumptions. Bull. World Health Organ..

[10] Mirambeau A.M., Guijing W., Ruggles L., Dunes O. (2013). A Cost Analysis of a Community Health Worker Program in Rural Vermont. J. Commun. Health.

[11] Walsh K., Levin H., Jaye P., Gazzard J. (2013). Cost analyses approaches in medical education: There are no simple solutions. Med. Educ..

[12] Walsh K., Donaldson S. (2010). Cost Effectiveness in Medical Education.

[13] Walsh K. (2016). Costs and assessment in medical education: A strategic view. Perspect. Med. Educ..

[14] Walsh K., Richardson J., Moore P. (2010). NICE medical education modules: An analysis of cost-effectiveness. Educ. Prim. Care.

[15] Vaughan K., Kok M.C., Witter S., Dieleman M. (2015). Costs and cost-effectiveness of community health workers: Evidence from a literature review. Hum. Resour. Health.

[16] McEwan P. (2012). Cost-effectiveness analysis of education and health interventions in developing countries. J. Dev. Eff..

[17] World Health Organization (2006). Working Together for Health.

[18] Bowser D., Okunogbe A., Oliveras E., Subramanian L., Morrill T. (2015). A Cost-Effectiveness Analysis of Community Health Workers in Mozambique. J. Prim. Care Commun. Health.

[19] Lindsay A. (1982). Institutional Performance in Higher Education. The Efficiency Dimension. Rev. Educ. Res..

[20] Psacharopoulos G., Patrinos H. (2018). Returns to Investment in Education. A Decennial Review of the Global Literature. Educ. Econ..

[21] Bouillon C., Tejerina L. (2016). Do We Know What Works? A Systematic Review of Impact Evaluations of Social Programs in Latin America and the Caribbean.

[22] Guisan M.C., Neira I. (2006). Direct and Indirect Effects of Human Capital on World Development, 1960–2004. Appl. Econom. Int. Dev..

[23] Barro R.J. (1996). Democracy and Growth. J. Econ. Growth.

[24] Gerring J., Bond P., Barndt W.T., Moreno C. (2005). Democracy and Economic Growth. A Historical Perspective. J. Political Econ..

[25] Ashenfelter O. (1978). Estimating the Effect of Training Programs on Earnings. Rev. Econ. Stat..

[26] Ruiz A.C., Gómez L.N., Narváez M.R. (2010). Endogenous Wage Determinants and Returns to Education in Spain. Int. J. Man Power.

[27] Schultz T. (1960). Capital Formation by Education. J. Political Econ..

[28] Weiss A. (1995). Human Capital vs. Signaling Explanations of Wages. J. Econ. Perspect..

[29] World Bank. www.Worldbank.org.

